# Supplementary material for: Reproducible research and GIScience: an evaluation using AGILE conference papers
Source: PeerJ. 2018 Jul 13;6:e5072. doi: 10.7717/peerj.5072 (PMC6047504; doi:10.7717/peerj.5072)
Supplement: Supplemental Information 1 [file peerj-06-5072-s001.pdf]

Paper Corpus for ”Reproducible research and GI-Science: an evaluation using AGILE conference papers”

Almer, A., R. Perko, H. Schrom-Feiertag, T. Schnabel, and L. Paletta (2016). **Critical Situation Monitoring at Large Scale Events from Airborne Video Based Crowd Dynamics Analysis**. In: *Geospatial Data in a Changing World*. Ed. by T. Sarjakoski, M. Y. Santos, and L. T. Sarjakoski. DOI: 10.1007/978-3-319-33783-8_20. Cham: Springer International Publishing, pp. 351–368. ISBN: 978-3-319-33783-8. URL: http://link.springer.com/10.1007/978-3-319-33783-8_20 (visited on 02/20/2018).

Baglatzi, A. and W. Kuhn (2013). **On the Formulation of Conceptual Spaces for Land Cover Classification Systems**. In: *Geographic Information Science at the Heart of Europe*. Ed. by D. Vandenbroucke, B. Bucher, and J. Crompvoets. DOI: 10.1007/978-3-319-00615-4_10. Cham: Springer International Publishing, pp. 173–188. ISBN: 978-3-319-00615-4. URL: http://link.springer.com/10.1007/978-3-319-00615-4_10 (visited on 02/20/2018).

Brinkhoff, T. (2017). **Supporting Dynamic Labeling in Web Map Applications**. In: *AGILE conference proceedings: Short Papers*. Wageningen, The Netherlands: Association of Geographic Information Laboratories in Europe (AGILE). URL: https://agile-online.org/conference_paper/cds/agile_2017/shortpapers/80_ShortPaper_in_PDF.pdf (visited on 02/20/2018).

Fan, H., A. Zipf, and Q. Fu (2014). **Estimation of Building Types on OpenStreetMap Based on Urban Morphology Analysis**. en. In: *Connecting a Digital Europe Through Location and Place*. Lecture Notes in Geoinformation and Cartography. DOI: 10.1007/978-3-319-03611-3_2. Springer, Cham, pp. 19–35. ISBN: 978-3-319-03611-3. URL: https://link.springer.com/chapter/10.1007/978-3-319-03611-3_2 (visited on 02/20/2018).

Foerster, T., B. Baranski, and H. Borsutzky (2012). **Live Geoinformation with Standardized Geoprocessing Services**. In: *Bridging the Geographic Information Sciences*. Ed. by J. Gensel, D. Josselin, and D. Vandenbroucke. DOI: 10.1007/978-3-642-29063-3_6. Berlin, Heidelberg: Springer Berlin Heidelberg, pp. 99–118. ISBN: 978-3-642-29063-3. URL: http://link.springer.com/10.1007/978-3-642-29063-3_6 (visited on 02/20/2018).

Fogliaroni, P. and H. Hobel (2014). **Linking crowdsourced observations with INSPIRE**. In: *AGILE conference proceedings: Short Papers*. Castellón, Spain: Association of Geographic Information Laboratories in Europe (AGILE). URL: https://agile-online.org/conference_paper/cds/agile_2014/agile2014_80.pdf (visited on 02/20/2018).

— (2015). **Implementing Naïve Geography via Qualitative Spatial Relation Queries**. In: *AGILE conference proceedings: Short Papers*. Lisbon, Portugal: Association of Geographic Information Laboratories in Europe

(AGILE). URL: https://agile-online.org/conference_paper/cds/agile_2015/shortpapers/91/91_Paper_in_PDF.pdf (visited on 02/20/2018).

Gröchenig, S., R. Brunauer, and K. Rehrl (2014). **Estimating Completeness of VGI Datasets by Analyzing Community Activity Over Time Periods**. In: *Connecting a Digital Europe Through Location and Place.* Ed. by J. Huerta, S. Schade, and C. Granell. DOI: 10.1007/978-3-319-03611-3_1. Cham: Springer International Publishing, pp. 3–18. ISBN: 978-3-319-03611-3. URL: http://link.springer.com/10.1007/978-3-319-03611-3_1 (visited on 02/20/2018).

Haumann, S. T., D. Bucher, and D. Jonietz (2017). **Energy-based Routing and Cruising Range Estimation for Electric Bicycles**. In: *AGILE conference proceedings: Short Papers.* Wageningen, The Netherlands: Association of Geographic Information Laboratories in Europe (AGILE). URL: https://agile-online.org/conference_paper/cds/agile_2017/shortpapers/145_ShortPaper_in_PDF.pdf (visited on 02/20/2018).

Heinz, T. and C. Schlieder (2015). **An Agent-Based Simulation Framework for Location-Based Games**. In: *AGILE conference proceedings: Short Papers.* Lisbon, Portugal: Association of Geographic Information Laboratories in Europe (AGILE). URL: https://agile-online.org/conference_paper/cds/agile_2015/shortpapers/114/114_Paper_in_PDF.pdf (visited on 02/20/2018).

Josselin, D., S. Boularouk, O. Bonin, and E. Altman (2016). **Sonorous Cartography for Sighted and Blind People**. In: *AGILE conference proceedings: Short Papers.* Helsinki, Finland: Association of Geographic Information Laboratories in Europe (AGILE). URL: https://agile-online.org/conference_paper/cds/agile_2016/shortpapers/124_Paper_in_PDF.pdf (visited on 02/20/2018).

Juhász, L. and H. H. Hochmair (2016). **Cross-Linkage Between Mapillary Street Level Photos and OSM Edits**. en. In: *Geospatial Data in a Changing World.* Lecture Notes in Geoinformation and Cartography. DOI: 10.1007/978-3-319-33783-8_9. Springer, Cham, pp. 141–156. ISBN: 978-3-319-33783-8. URL: https://link.springer.com/chapter/10.1007/978-3-319-33783-8_9 (visited on 02/20/2018).

Kiefer, P., F. Straub, and M. Raubal (2012). **Location-Aware Mobile Eye-Tracking for the Explanation of Wayfinding Behavior**. In: *AGILE conference proceedings: Short Papers.* Avignon, France: Association of Geographic Information Laboratories in Europe (AGILE). URL: https://agile-online.org/conference_paper/cds/agile_2012/proceedings/papers/paper_kiefer_location-aware_mobile_eye-tracking_for_the_explanation_of_wayfinding_behavior_2012.pdf (visited on 02/20/2018).

Knoth, L., M. Mittlboeck, and B. Vockner (2017). **3D Building Maps for Everyone—Mapping Buildings Using VGI**. In: *Societal Geo-innovation.* Ed. by A. Bregt, T. Sarjakoski, R. van Lammeren, and F. Rip. DOI: 10.1007/978-3-319-56759-4_5. Cham: Springer International Publishing, pp. 77–91. ISBN: 978-3-319-56759-4. URL: http://link.springer.com/10.1007/978-3-319-56759-4_5 (visited on 02/20/2018).

Konkol, M., C. Kray, and M. Ostkamp (2017). **Follow the Signs—Countering Disengagement from the Real World During City Exploration**. en. In: *Societal Geo-innovation*. Lecture Notes in Geoinformation and Cartography. Springer, Cham, pp. 93–109. ISBN: 978-3-319-56759-4. DOI: 10.1007/978-3-319-56759-4_6. URL: https://link.springer.com/chapter/10.1007/978-3-319-56759-4_6 (visited on 02/20/2018).

Körner, C., D. Hecker, M. May, and S. Wrobel (2010). **Visit Potential: A Common Vocabulary for the Analysis of Entity-Location Interactions in Mobility Applications**. In: *Geospatial Thinking*. Ed. by M. Painho, M. Y. Santos, and H. Pundt. Vol. 0. DOI: 10.1007/978-3-642-12326-9_5. Berlin, Heidelberg: Springer Berlin Heidelberg, pp. 79–95. ISBN: 978-3-642-12326-9. URL: http://link.springer.com/10.1007/978-3-642-12326-9_5 (visited on 02/20/2018).

Kuhn, W. and A. Ballatore (2015). **Designing a Language for Spatial Computing**. In: *AGILE 2015*. Ed. by F. Bacao, M. Y. Santos, and M. Painho. DOI: 10.1007/978-3-319-16787-9_18. Cham: Springer International Publishing, pp. 309–326. ISBN: 978-3-319-16787-9. URL: http://link.springer.com/10.1007/978-3-319-16787-9_18 (visited on 02/20/2018).

Magalhães, S. V. G., M. V. A. Andrade, W. Randolph Franklin, and G. C. Pena (2012). **A New Method for Computing the Drainage Network Based on Raising the Level of an Ocean Surrounding the Terrain**. In: *Bridging the Geographic Information Sciences*. Ed. by J. Gensel, D. Josselin, and D. Vandenbroucke. DOI: 10.1007/978-3-642-29063-3_21. Berlin, Heidelberg: Springer Berlin Heidelberg, pp. 391–407. ISBN: 978-3-642-29063-3. URL: http://link.springer.com/10.1007/978-3-642-29063-3_21 (visited on 02/20/2018).

Mazimpaka, J. D. and S. Timpf (2015). **Exploring the Potential of Combining Taxi GPS and Flickr Data for Discovering Functional Regions**. In: *AGILE 2015*. Ed. by F. Bacao, M. Y. Santos, and M. Painho. DOI: 10.1007/978-3-319-16787-9_1. Cham: Springer International Publishing, pp. 3–18. ISBN: 978-3-319-16787-9. URL: http://link.springer.com/10.1007/978-3-319-16787-9_1 (visited on 02/20/2018).

Merki, M. and P. Laube (2012). **Detecting reaction movement patterns in trajectory data**. In: *AGILE conference proceedings: Short Papers*. Avignon, France: Association of Geographic Information Laboratories in Europe (AGILE). URL: http://www.geo.uzh.ch/~plaube/pubs/merkiLaubeAgile12.pdf (visited on 02/20/2018).

Osaragi, T. and T. Hoshino (2012). **Predicting Spatiotemporal Distribution of Transient Occupants in Urban Areas**. In: *Bridging the Geographic Information Sciences*. Ed. by J. Gensel, D. Josselin, and D. Vandenbroucke. DOI: 10.1007/978-3-642-29063-3_17. Berlin, Heidelberg: Springer Berlin Heidelberg, pp. 307–325. ISBN: 978-3-642-29063-3. URL: http://link.springer.com/10.1007/978-3-642-29063-3_17 (visited on 02/20/2018).

Osaragi, T. and S. Tsuda (2013). **Facility Use-Choice Model with Travel Costs Incorporating Means of Transportation and Travel Direction**. In: *Geographic Information Science at the Heart of Europe*. Ed. by

D. Vandenbroucke, B. Bucher, and J. Crompvoets. DOI: 10.1007/978-3-319-00615-4_17. Cham: Springer International Publishing, pp. 307–322. ISBN: 978-3-319-00615-4. URL: http://link.springer.com/10.1007/978-3-319-00615-4_17 (visited on 02/20/2018).

Raubal, M. and S. Winter (2010). **A Spatio-Temporal Model Towards Ad-Hoc Collaborative Decision-Making**. In: *Geospatial Thinking*. Ed. by M. Painho, M. Y. Santos, and H. Pundt. Vol. 0. DOI: 10.1007/978-3-642-12326-9_15. Berlin, Heidelberg: Springer Berlin Heidelberg, pp. 279–297. ISBN: 978-3-642-12326-9. URL: http://link.springer.com/10.1007/978-3-642-12326-9_15 (visited on 02/20/2018).

Rosser, J., A. Pourabdollah, R. Brackin, M. Jackson, and D. G. Leibovici (2016). **Full Meta Objects for flexible geoprocessing workflows: profiling WPS or BPMN?** In: *AGILE conference proceedings: Short Papers*. Helsinki, Finland: Association of Geographic Information Laboratories in Europe (AGILE). URL: https://agile-online.org/conference_paper/cds/agile_2016/shortpapers/160_Paper_in_PDF.pdf (visited on 02/20/2018).

Schäffer, B., B. Baranski, and T. Foerster (2010). **Towards Spatial Data Infrastructures in the Clouds**. In: *Geospatial Thinking*. Ed. by M. Painho, M. Y. Santos, and H. Pundt. Vol. 0. DOI: 10.1007/978-3-642-12326-9_21. Berlin, Heidelberg: Springer Berlin Heidelberg, pp. 399–418. ISBN: 978-3-642-12326-9. URL: http://link.springer.com/10.1007/978-3-642-12326-9_21 (visited on 02/20/2018).

Scheider, S., J. Jones, A. Sánchez, and C. Keßler (2014). **Encoding and Querying Historic Map Content**. In: *Connecting a Digital Europe Through Location and Place*. Ed. by J. Huerta, S. Schade, and C. Granell. DOI: 10.1007/978-3-319-03611-3_15. Cham: Springer International Publishing, pp. 251–273. ISBN: 978-3-319-03611-3. URL: http://link.springer.com/10.1007/978-3-319-03611-3_15 (visited on 02/20/2018).

Schwering, A., R. Li, and V. J. A. Anacta (2013). **Orientation Information in Different Forms of Route Instructions**. In: *AGILE conference proceedings: Short Papers*. Leuven, Belgium: Association of Geographic Information Laboratories in Europe (AGILE). URL: https://agile-online.org/conference_paper/cds/agile_2013/short_papers/sp_sbp_li.pdf (visited on 02/20/2018).

Soleymani, A., E. E. van Loon, and R. Weibel (2014). **Capability of movement features extracted from GPS trajectories for the classification of fine-grained behaviors**. eng. In: *AGILE conference proceedings: Short Papers*. Castellón, Spain: Association of Geographic Information Laboratories in Europe (AGILE). ISBN: 978-90-816960-4-3. DOI: 10.5167/uzh-101009. URL: http://www.agile-online.org/Conference_Paper/cds/agile_2014/agile2014_150.pdf (visited on 02/20/2018).

Stein, K. and C. Schlieder (2012). **A Geowiki for Participatory Mobility**. In: *AGILE conference proceedings: Short Papers*. Avignon, France: Association of Geographic Information Laboratories in Europe (AGILE). URL: https:

//agile-online.org/conference_paper/cds/agile_2013/short_papers/sp_sbp_stein.pdf (visited on 02/20/2018).

Steuer, H., T. Machl, M. Sindram, L. Liebel, and T. H. Kolbe (2015). **Voluminator—Approximating the Volume of 3D Buildings to Overcome Topological Errors**. en. In: *AGILE 2015*. Lecture Notes in Geoinformation and Cartography. DOI: 10.1007/978-3-319-16787-9_20. Springer, Cham, pp. 343–362. ISBN: 978-3-319-16787-9. URL: https://link.springer.com/chapter/10.1007/978-3-319-16787-9_20 (visited on 02/20/2018).

Wiemann, S. (2016). **Spatial Data Relations as a Means to Enrich Species Observations from Crowdsourcing**. In: *Geospatial Data in a Changing World*. Ed. by T. Sarjakoski, M. Y. Santos, and L. T. Sarjakoski. DOI: 10.1007/978-3-319-33783-8_8. Cham: Springer International Publishing, pp. 123–140. ISBN: 978-3-319-33783-8. URL: http://link.springer.com/10.1007/978-3-319-33783-8_8 (visited on 02/20/2018).

Zhu, R., P. C. Kyriakidis, and K. Janowicz (2017). **Beyond Pairs: Generalizing the Geo-dipole for Quantifying Spatial Patterns in Geographic Fields**. In: *Societal Geo-innovation*. Ed. by A. Bregt, T. Sarjakoski, R. van Lammeren, and F. Rip. DOI: 10.1007/978-3-319-56759-4_19. Cham: Springer International Publishing, pp. 331–348. ISBN: 978-3-319-56759-4. URL: http://link.springer.com/10.1007/978-3-319-56759-4_19 (visited on 02/20/2018).
