# Supplementary material for: Reproducible research and GIScience: an evaluation using AGILE conference papers
Source: PeerJ. 2018 Jul 13;6:e5072. doi: 10.7717/peerj.5072 (PMC6047504; doi:10.7717/peerj.5072)
Supplement: Supplemental Information 2 [file peerj-06-5072-s002.pdf]

Reproducible Research @ AGILE

The term Reproducible Research refers to the idea that the ultimate product of academic research is a scientific paper published along with the data, methods and full computational environment used to produce the results in the paper.

Reproducibility is a principle of good scientific practice that allows other researchers to verify work that has been done. We are interested in the current state of reproducibility in GIScience. Our motivation is to develop concrete recommendations for improving reproducibility of research work in the GIScience community.

Your views and feedback on the reproducibility of your research would be highly appreciated!

1. **Email address** *

____

2. **Please select your nominated AGILE Best Paper.**
Mark only one oval.

- ◯ Zhu et al. (2017) "Beyond pairs: Generalizing the geo-dipole for quantifying spatial patterns in geographic fields"
- ◯ Knoth et al. (2017) "3D Building Maps for Everyone - Mapping Buildings Using VGI"
- ◯ Konkol et al. (2017) "Follow the Signs Countering Disengagement from the Real World During City Exploration"
- ◯ Haumann et al. (2017) "Energy-based Routing and Cruising Range Estimation for Electric Bicycles"
- ◯ Brinkhoff (2017) "Supporting Dynamic Labeling in Web Map Applications"
- ◯ Almer et al. (2016) "Critical Situation Monitoring at Large Scale Events from Airborne Video Based Crowd Dynamics Analysis Alexander"
- ◯ Wiemann (2016) "Spatial Data Relations as a Means to Enrich Species Observations from Crowdsourcing"
- ◯ Juhasz & Hochmair (2016) "Cross-Linkage Between Mapillary Street Level Photos and OSM Edits"
- ◯ Josselin et al. (2016) "Sonorous Cartography for Sighted and Blind People"
- ◯ Rosser et al. (2016) "Full Meta Objects for flexible geoprocessing workflows: profiling WPS or BPMN?"
- ◯ Kuhn & Ballatore (2015) "Designing a Language for Spatial Computing"
- ◯ Mazimpaka & Timpf (2015) "Exploring the Potential of Combining Taxi GPS and Flickr Data for Discovering Functional Regions"
- ◯ Steuer et al. (2015) "Voluminator—Approximating the Volume of 3D Buildings to Overcome Topological Errors"
- ◯ Fogliaroni & Hobel (2015) "Implementing Naïve Geography via Qualitative Spatial Relation Queries"
- ◯ Heinz & Schlieder (2015) "An Agent - Based Simulation Framework for Location - Based Games"
- ◯ Scheider et al. (2014) "Encoding and Querying Historic Map Content"
- ◯ Groechenig et al. (2014) "Estimating completeness of VGI datasets by analyzing community activity over time periods"
- ◯ Fan et al. (2014) "Estimation of Building Types on OpenStreetMap Based on Urban Morphology Analysis Hongchao"
- ◯ Soleymani et al. (2014) "Capability of movement features extracted from GPS trajectories for the classification of fine-grained behaviors"
- ◯ Wiemann & Bernard (2014) "Linking crowdsourced observations with INSPIRE"
- ◯ Osaragi & Tsuda (2013) "Facility use-choice model with travel costs incorporating means of transportation and travel direction"
- ◯ Baglatzi & Kuhn (2013) "On the formulation of conceptual spaces for land cover classification systems"
- ◯ Schwering et al. (2013) "Orientation Information in Different Forms of Route Instructions"
- ◯ Stein & Schlieder (2013) "A Geowiki for Participatory Mobility 2 Volunteered Geographic Information"
- ◯ Osaragi & Hoshino (2012) "Predicting Spatiotemporal Distribution of Transient Occupants in Urban Areas"
- ◯ Magalhaes et al. (2012) "A new method for computing the drainage network based on raising the level of an ocean surrounding the terrain"
- ◯ Foerster et al. (2012) "Live geoinformation with standardized geoprocessing services"
- ◯ Merki & Laube (2012) "Detecting reaction movement patterns in trajectory data"

- Kiefer et al. (2012) "Location-Aware Mobile Eye-Tracking for the Explanation of Wayfinding behavior"
- Raubal & Winter (2010) "A spatio-temporal model towards ad-hoc collaborative decision-making"
- Schaeffer et al. (2010) "Towards Spatial Data Infrastructures in the Clouds"
- Korner et al. (2010) "Visit Potential: A Common Vocabulary for the Analysis of Entity-Location Interactions in Mobility Applications"

3. **Are you the first author of this paper?**
   Mark only one oval.

   - Yes
   - No

4. **Have you considered the reproducibility of research published in your nominated paper?**
   Mark only one oval.

   - Yes, it is important to me that my research is fully reproducible
   - Yes, I have somewhat considered reproducibility
   - No, I was not concerned with it
   - Other: ____________________

We have investigated all the papers nominated for the AGILE Best Paper in 2010-2017. The following table shows the results of our evaluation. You can find your paper there as well.

	Data	Methods			Results
		Data Preprocessing	Algorithms	Computational Environment	
Zhu et al. (2017)	0	1	1	1	1
Knoth et al. (2017)	0	x	0	1	1
Konkol et al. (2017)	2	2	1	2	1
Haumann et al. (2017)	0	1	1	0	1
Brinkhoff (2017)	0	x	1	0	0
Almer et al. (2016)	0	x	1	1	1
Wiemann (2016)	2	x	1	1	1
Juhasz & Hochmair (2016)	0	1	1	0	0
Josselin et al. (2016)	1	x	0	0	1
Rosser et al. (2016)	0	x	1	1	0
Kuhn & Ballatore (2015)	x	x	1	0	1
Mazimpaka & Timpf (2015)	2	1	1	1	1
Steuer et al. (2015)	2	0	1	1	1
Fogliaroni & Hobel (2015)	x	x	x	x	x
Heinz & Schlieder (2015)	0	0	1	1	1
Scheider et al. (2014)	1	1	2	1	1
Groechenig et al. (2014)	2	0	1	0	1
Fan et al. (2014)	0	1	1	0	1
Soleymani et al. (2014)	0	0	1	0	0
Wiemann & Bernard (2014)	0	0	1	0	0
Osaragi & Tsuda (2013)	0	1	1	0	1
Baglatzi & Kuhn (2013)	x	x	x	x	x
Schwering et al. (2013)	0	0	1	x	1
Stein & Schlieder (2013)	0	x	1	0	1
Osaragi & Hoshino (2012)	0	0	1	0	1
Magalhaes et al. (2012)	0	0	1	0	0
Foerster et al. (2012)	1	x	1	1	1
Merki & Laube (2012)	0	0	0	0	0
Kiefer et al. (2012)	0	1	1	0	1
Raubal & Winter (2010)	x	x	x	x	x
Schaeffer et al. (2010)	0	0	1	2	1
Korner et al. (2010)	x	x	x	x	x
Kuhn & Raubal (2003)	x	x	2	1	1

code explanation

- 0 unavailable (including available upon request) and not recreatable
- 1 documented and recreatable
- 2 available, but with non-public licenses/no license or non-permanent websites
- 3 available, open and permanent (with DOI)
- x not applicable

5. **Do you agree with our rating? Please comment**

Not too surprisingly, we have found that none of the papers were fully reproducible*.

*a fully reproducible paper would make sure that a reviewer or reader can recreate precisely the setting, including the prerequisite knowledge and the computational environment, of a research paper. In case of the former, the argument must be clear and understandable, the latter requires a detailed description of used software and data, both of which must be openly available.

6. **Please rate how strongly the following circumstances have hindered you from providing all data, methods and results used/developed during your research?**

Mark only one oval per row.

	Not at all	Slightly hindered	Moderately hindered	Strongly hindered	Main reason
The need to invest more time into the publication					
Lack of knowledge how to include data/methods/results into the publication					
Lack of tools that would help to attach data/methods/results to the publication					
Lack of motivation or incentive					
Legal restrictions (e.g. privacy issues or copyright)					

7. **Please add here if there were any other hindering circumstances**

8. **What would you suggest to AGILE community to encourage publishing fully reproducible papers?**

9. **We would like to ask for your consent to discuss your answers to this survey in our publication.**

Check all that apply.

- [ ] Yes, I agree that my answers will be discussed in a publication.

- [ ] Send me a copy of my responses.

Powered by
Google Forms
